# Major depressive disorder (MDD) in adolescents with Borderline (BPD) and Shyzotypal personality disorder (STPD)

**DOI:** 10.1192/j.eurpsy.2023.1571

**Published:** 2023-07-19

**Authors:** V. Kaleda, E. Krylova, A. Kuleshov

**Affiliations:** Department of youth psychiatry, Federal State Budgetary Scientific Institution Mental Health Research Center, Moscow, Russian Federation

## Abstract

**Introduction:**

MDD is a chronic illness characterized by complex patterns of persistence, remission and relapse. Personality disorder (PD) is a negative prognostic marker MDD and its chronicity, and dynamics of STPD and BPD in adolescence occurs most severely.

**Objectives:**

The purpose of this study was to study the effect of STPD and BPD on the course of MDD in comparative groups in youth.

**Methods:**

Clinical psychopathological interview and follow-up assessment, SCID-II, Hamilton Depression Rating Scale (HDRS). Sample: N=102 male and female with MDD in STPD and BPD into two equal groups of 51 people respectively. MDD was assessed in each of both PD groups in two time frames: current and during depressive the last 12 months in adolescence; three and more years later during adolescence. Outcome predictors included: clinical characteristics of MDD, duration of current episode, relapse rate. The average age of first MDD in both groups was 18.9±3.2 years.

**Results:**

The first MDD with both PD in adolescence characterized by psychopatological variety due to type PD and age factor. Depressive was more variability of affect, and shorter duration (less then 6 months N=28 (55%)) in group BPD as opposed to the sustained mood problems seen in STPD – (6-12 months N=19 (37%); 12 months and more N=21(41%)) with MDD (p=0,004; χ2= 14,997). During the subsequent follow-up assessment among patients with BPD had the highest percent recurrences once every 6 months or more (N=36 (71%)) and those with STPD significantly rarer recurrences, every 6 to 12 months (N=21(41%)), Less than once a year N=17 (33%)), (p=0,001; χ2= 23,252). Diagnosis of PD in adolescence is based on pathological traits, and impairment in the affective disorder is measurement separately. When measuring impulsivity in the BPD group, significantly higher rates BIS-11 scale were noted (74±1) than in the SPD group (61±1).

**Conclusions:**

Diagnosis of PD in adolescence is based on pathological traits, and impairment in the affective disorder is measurement separately. It has been confirmed that PDs are a negative prognostic marker of the manifestation of a MDD in adolescence, and the presence of BPD is a serious prognostic predictor of its persistence.

**Disclosure of Interest:**

None Declared

